# Assessing the performance of environmental management in academic research laboratories^☆^

**DOI:** 10.1016/j.heliyon.2022.e09135

**Published:** 2022-03-21

**Authors:** M. Ladyman, E. Gutierrez-Carazo, F. Persico, T. Temple, F. Coulon

**Affiliations:** a Cranfield University, Centre for Defence Chemistry, Defence Academy of the United Kingdom, Shrivenham SN6 7LA, UK; b Cranfield University, School of Water, Energy and Environment, Cranfield MK43 0AL, UK

**Keywords:** Environmental performance, Plan-do-check-act, Deming cycle, Environmental hazard, Environmental questionnaire

## Abstract

Managing environmental risk is essential to ensure organisations minimise their impact on the environment, comply with environmental legislation and maintain their reputation in an increasingly environmentally aware society. Organisations frequently use management systems to plan and execute routine environmental assessments, however environmental impacts may still arise from routine activities or accidents that could be avoided by effective environmental management. Currently there is no method for an organisation to assess the level of awareness their employees have of activities that may lead to an environmental impact, or the level of uptake of environmental management processes. Therefore, the Environmental Management Performance Assessment (EMPA) process was developed to enable organisations to self-assess existing environmental management processes by survey of their employees. The EMPA process was aligned to key phases of the Deming Cycle and involves development and distribution of a survey to organisation employees. The responses are then used to recognise areas for improvement by progression through a bespoke flow chart integrated with the initial survey. This enables demonstration of how particular hazards arise from insufficient awareness at different stages in the Deming Cycle and how these hazards can have wider, reputational, economic, and legislative consequences. The process was trialled by surveying academic researchers on the environmental management processes in their laboratories as a sample set.

## Graphical abstract

**Figure:**
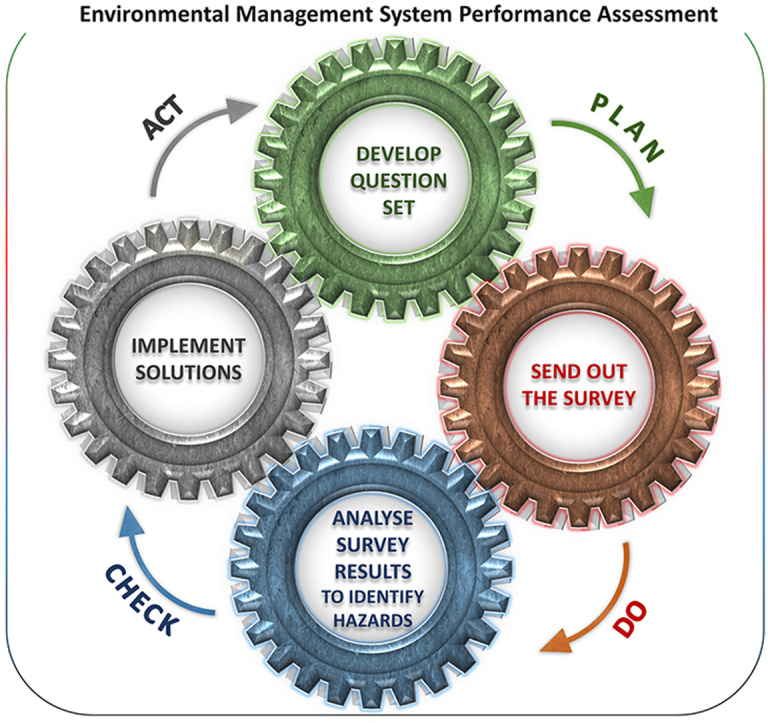

## 1 Introduction

Environmental risk is defined as the actual or potential threat of adverse effects on living organisms and ecosystems from anthropogenic activities [1]. In general terms, environmental risk depends on three factors, the levels of unwanted contaminant or nuisances in the environment, the exposure of receptors to the contaminant or nuisance, and the effect a contaminant or nuisance has on the receptor e.g., toxicity or level of harm [1]. The effect on the environment can be from the discharge of effluents, emissions, wastes or the use of scarce resources arising from any organisation's activities such as offices, manufacturing facilities and academic research laboratories [2]. To manage these impacts an Environmental Risk Assessment (ERA) can be used. This is outlined in codes of practice guidance such as, British Standard BS 31100, which includes the overall process of environmental risk identification, evaluation, and mitigation [3].

**Figure 1:**
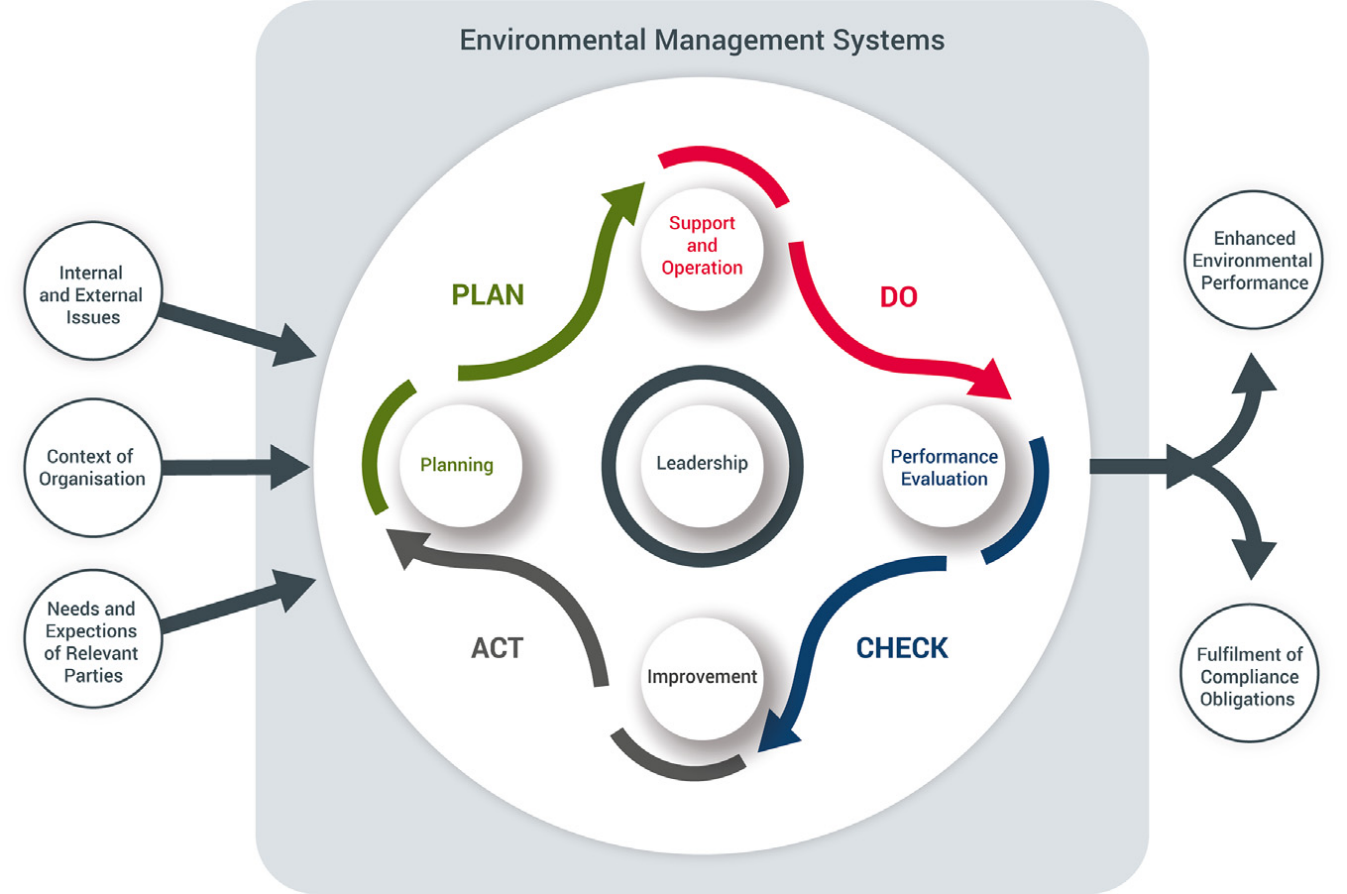
Outline of Environmental Management System process, with the Deming Cycle aligned to key phases of ISO14001:2015 (leadership, support and operation, performance evaluation, improvement, and planning). An example of how an EMS embeds plan-do-check-act.

To achieve effective ERA, organisations often apply environmental management strategies [4]. Environmental management helps to ensure that environmental risk is contained to acceptable levels and ideally should be applied to all aspects of an organisation by supporting the inclusion of relevant environmental legislation, internal and external stakeholder engagement and cost-effective mitigation [5]. An example of an internationally accepted environmental management standard is the International Organisation for Standardisation (ISO) Environmental Management Systems, ISO 14001. ISO 14001 [5] like most management systems includes the Plan-Do-Check-Act continuous improvement mechanism, known as the Deming Cycle [6]. The plan-do-check-act elements mean an organisation must first develop a workable plan to meet the requirement, e.g., understanding the context of the organisation, identify relevant stakeholders, define the scope of the implementation, and determine how the system will be managed and maintained. The do-phase involves putting the plan in place, establishing the ERA methodologies, and making sure they are carried out and include effective mitigation. Checking that the Environmental Management System (EMS) is working efficiently by reviewing continuously the entire systems and ensuring progress against the objectives is the third step. The act phase involves addressing any issues in the check phase prior to the next iteration of the cycle, thus ensuring continual improvement (Figure 1).

The EMS process is designed to capture all environmental obligations of an organisation, and to promote best practice to ensure environmental compliance, staff awareness and ultimately minimise environmental impact. In recent years many organisations have implemented environmental management processes such as ISO14001, carbon foot-printing or lifecycle assessment to demonstrate their compliance with legislation and meet national and international environmental objectives [7, 8]. However, even with such systems in place environmental impacts have been realised due to accidents or inefficient management of routine activities [9]. For example, in the University of Lyon (France), a fireball erupted into the sky above one of the buildings due to a gas bottle explosion. Although the authorities at the university insisted there were no risks of chemical leak, a massive plume of black smoke and flickering flames was observed, indicating a release of toxic gases that could affect the environment [10].

At Beijing Jiaotong University (China), during sewage treatment experiments, hydrogen generated by a reaction between pyrophosphoric acid and magnesium was ignited by a metal-friction spark. The resulting explosion provoked a second magnesium dust explosion that trapped the rest of the magnesium powder and other combustibles nearby and released toxins into the environment [11]. Other uncontrolled explosions have been reported in the USA, in the University of California at Los Angeles and in the Chemistry and Biochemistry Department at Texas. They presented similar environmental consequences, however in these cases the accidents also affected human health [12, 13].

However, the environmental consequences of incidents can be successfully avoided using ERA and effectively communicating the contents to all stakeholders. For example, at the University of Bristol, a chemistry building was evacuated after a student accidently synthesized an explosive compound. The student had identified that there was a risk of the explosive being created as a potential by-product while conducting the risk assessment and Control of Substances Hazardous to Health (COSHH), therefore recognised the risk immediately and was able to prevent harm by notifying those responsible for lab safety [14]. The laboratory was evacuated safely, and the explosive disposed in-line with mitigation that was recommended by the initial risk assessment. A similar case occurred in Manchester University where a hazardous compound (explosion risk), acetone peroxide, was accidently synthesized. Due to the prior completion of ERA the researchers were able to recognise when the compound had been synthesised and could act before any danger was caused by calling the emergency services [15].

In both these cases, it is clear that engaging the researchers themselves in the environmental risk assessment process was essential to the successful management of the incidents [16]. The researchers were able to respond rapidly, and report immediately to the responsible persons, ultimately avoiding any major environmental impacts. Research on implementation of EMS at European academic institutions has shown that a participatory approach which includes staff and students in the process improves environmental performance [3]. In addition, many management systems, including ISO14001, recommend the inclusion of the employees who undertake the activities in the risk assessment and management process to ensure that all potentially environmentally damaging activities are captured, and that the subsequent management or mitigation processes are followed [17].

However, it can be challenging to assess the level of uptake of proposed environmental management processes by employees, and to determine why certain processes may not be effective. For example, while an organisation may have implemented an EMS, if this is not communicated to employees as routine procedure or through awareness courses or specific training, the proposed mitigations may not be carried out [18]. Similarly, if employees have no mechanism for reporting environmental issues to higher management, the EMS may not be able to continually improve [19]. To implement continual improvement mechanisms one method is to monitor key performance indicators [20, 21], such as percentage reduction in carbon emissions, and while this is a barometer for environmental performance it cannot identify where practical improvements to an EMS can be implemented. Internal audits are also a valuable tool, but these tend to be a snapshot of activity and cannot provide information on how EMS processes are being communicated and undertaken by staff and may also be resource intensive for an organisation. It has also been shown that employee perception of environmental performance can be a useful way to identify areas for improvement within organisations using questionnaires and surveys [22]. Therefore, the aim of this work was to develop a method for organisations to self-assess the performance, suitability, and employee awareness of Environmental Management processes to identify areas for improvement that without management may lead to negative environmental impact. Academic research institutes were used as an example organisation to demonstrate the Environmental Management Performance Assessment (EMPA) process.

## 2 Method

EMPA was designed to systematically assess environmental performance and identify areas for improvement of environmental risk management in an organisation by mapping survey responses to the EMPA flowchart. The following sections outline how the EMPA flowchart was created, how the survey was structured and how this was then applied specifically to academic research institutions. Academic researchers were selected as the sample set as they are likely to be working in laboratories where their activities have the potential to create environmental impact if not managed correctly.

### 2.1 Environmental management systems performance assessment flowchart development

The EMPA flowchart was created by developing a series of binary steps aligned to the ISO14001 audit question set linked to the Deming Cycle to represent the processes within an EMS (Table 1). Surveying researchers across a global range of academic institutions provided evidence of whether any environmental management processes have been effectively communicated and implemented and are continually improved over time. Progression through the flowchart is through an affirmative or negative pathway. Affirmative responses enable advancement through the flowchart to the next phase in the Deming cycle, and negative responses reveal the hazard toward the efficacy of environmental management. Therefore, when moving through the flowchart if the answer to a binary step is negative, the pathway ends at an identified hazard indicating that this step is an area for improvement (Table 1).

**Table 1:** Flowchart steps aligned to key phases of ISO14001:2015 audit question set and the deming cycle.

Flowchart Steps	Alignment with ISO14001	Hazard
1: Does your organisation require you to be aware of the environmental risk of your research? *Questions specified whether researchers had to acknowledge or write an environmental risk assessment.*	**Plan** – Establish, implement, and maintain the processes needed to meet the requirements.	Lack of engagement *The researchers have no knowledge of the EMS, or there is no EMS in place*.
2: On a scale of 1–5 how often do you consider the environmental impact of your research? *1= never; 5= always.*	**NA**- Inquiry into personal values of respondents.	
3: Where is your organisation (UK, EU, North America, South America, Asia, Australasia, Africa)?	**NA**- Demographic question.	
4: Does your organization provide ERA training? *Two related questions asked whether the organisation provided environmental awareness training or ERA training. Answers were combined.*	**Plan**- the organisation shall establish, implement, and maintain the process(es) needed to meet the requirements.	Ineffective communication *An EMS is in place, but researchers are unaware of what it involved and have no awareness of training etc...*
5: Do you know who is responsible for your ERA? *‘Yes’ responses, and respondents who gave a free text response naming a role were combined for ‘yes’.*	**Do** - Determine and provide the resources needed for the establishment, implementation, maintenance, and continual improvement of the EMS.	No feedback Mechanism *An EMS is established and risk assessments for research projects are frequently implemented but researchers are unaware of how to communicate mitigations or changes to the process.*
6: Do you know who to report an environmental accident to? *‘No’ and ‘unsure’ responses were combined.*	**Do**- Operational planning and control.	
7: Does your ERA require authorization? *Responses combined with step 8.*	**Do** - Operational planning and control.	
8: Is the ERA reviewed regularly? *‘No’ and ‘unsure’ responses were combined.*	**Check** – Performance evaluation.	
9: Are the completed ERAs available organization-wide? *Response options were ‘stored locally- digitally and/or hard copy’, ‘stored on a database’ and ‘other’. Respondents who ticked ‘other’ were asked to provide more details in a free text box.*	**Act** - Management review.	No continual Improvement *An EMS is established and risk assessments for research projects are frequently implemented and reviewed.*

### 2.2 Environmental management systems performance assessment survey

To trial EMPA an electronic survey was created and distributed to laboratory researchers using the online Qualtrics XM survey tool [23]. The survey was designed to be concise with a total of 16 forced-single-choice questions, 1 multi-choice and 3 ‘open-answer’. The survey questions were developed based on the flowchart and were written to obscure their link to the Deming Cycle and ISO14001 so that responses were non-biased [24] (Table 1). The main criteria for respondents taking the survey were that they had undertaken research in a university laboratory either as a student or staff member within the last five years. The survey was designed to filter the respondents as they answered questions e.g., if a respondent was not aware of a need to actively undertake an ERA, they would not be required to answer the questions on specific environmental management processes. The questions were structured to enable respondents to exit if they were not able to answer the more specific questions in the survey, these were then recorded as a negative response. The survey was conducted anonymously, and the only demographic questions were to record their geographic location and their laboratory role.

The survey was designed to be completed within approximately 5 minutes, although this was dependent on extra time taken when answering the ‘open-answers’. The term Environmental Risk was defined prior the beginning of the survey as ‘the actual or potential threat of adverse effects on living organisms and the environment by effluents, emissions, wastes, resource depletion, etc., arising out of an organisation's activities’ [2]. The survey was distributed via on-line platforms, including LinkedIn, ResearchGate and Twitter and was active for a total of 47 days. Approval for the survey was granted through from the Cranfield University Research Ethics System (CURES) and informed consent was obtained for all participants prior to completion of the survey (Supplementary). The survey received 171 responses.

### 2.3 Environmental risk management assessment of research laboratories

Survey results were extracted from the Qualtrics XM software and mapped against the pathways in the EMPA flowchart as a percentage of affirmative or negative responses for each step. This enabled further analysis of the hazards to extrapolate consequences for a university if environmental management processes were absent or inefficiently implemented.

## 3 Results and discussion

**Figure 2:**
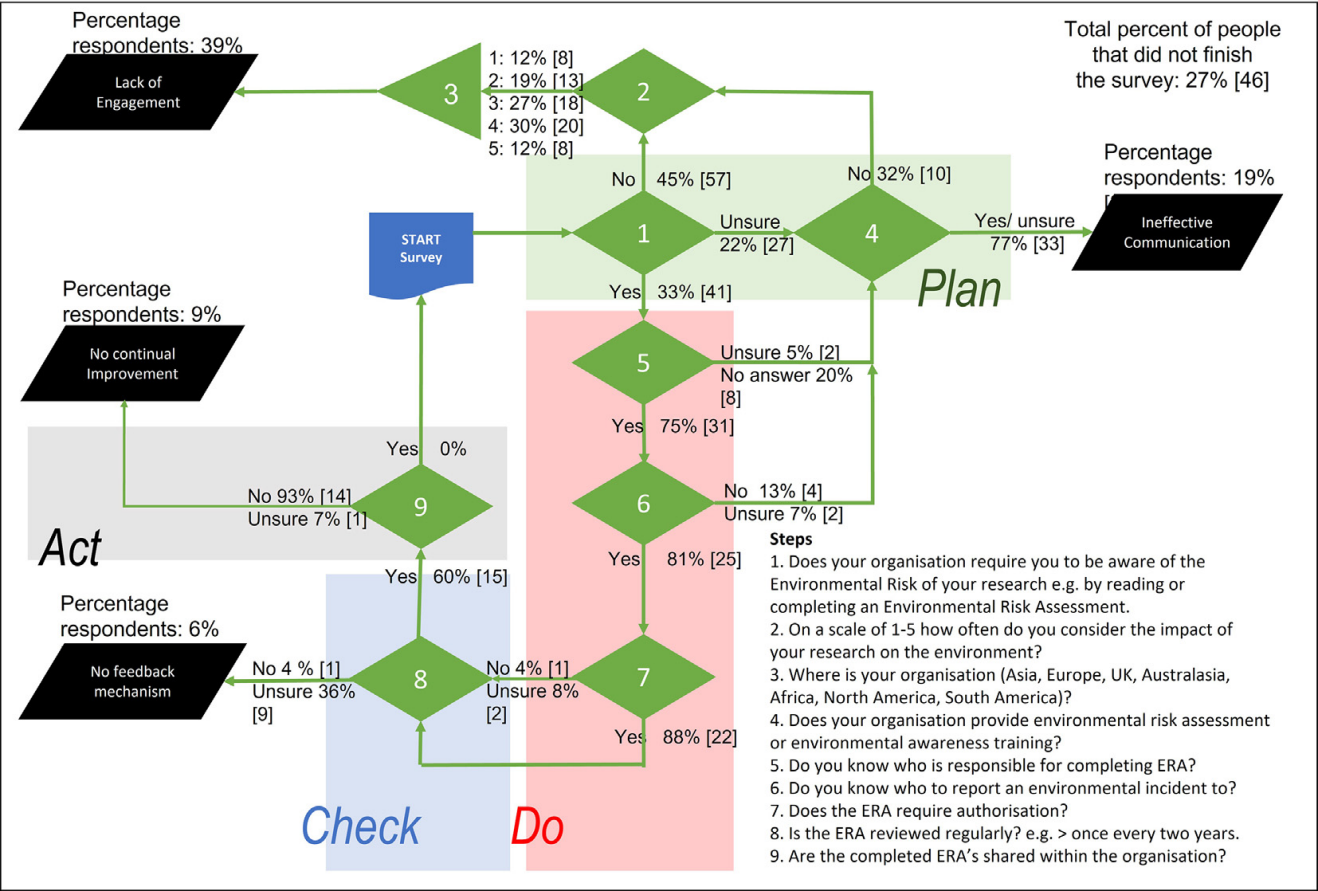
Environmental Management Systems Performance Assessment (EMPA) flowchart developed to align to ISO14001 and Deming Cycle (plan-do-check-act) demonstrating the pathways (green) and endpoints (hazards) (black) and the percentage responses for each question. The start point is at the blue box.

As an example of EMPA, academic research institutions were selected as laboratory work has the potential to cause high environmental impacts. For example, some research equipment may use significant quantities of electricity *i*.e., 10% or more of the total university demand. If this is not captured by a project specific ERA, the opportunity to manage or reduce energy usage may be missed. This means, that within a university EMS processes need to be communicated to researchers so that appropriate management and mitigation can be implemented, and to ensure that wider initiatives for continual improvement are developed. In addition, by surveying the researchers themselves on their understanding of the EMS it is possible to gather evidence for how efficiently the EMS is being communicated and monitored throughout the academic institution. The overall results from the survey suggest that globally environmental management is inconsistent, with 46% of respondents coming from organisations that do not have an EMS. The following sections will use the survey results to describe the pathways through the EMPA flowchart (Figure 2).

Steps 1 to 4 of the EMPA flowchart link to the ‘planning’ stage of the Deming Cycle and a high percentage of negative responses from these steps indicate that there is not significant engagement with EMS. In some countries, this may be due to the level of environmental regulation, which may be minimal in some areas. However, compliance should not be the only reason to undertake environmental management, and where there is not a legal mandate, benefits may be accrued from applying an environmental management plan such as cost savings and increased reputation. Where environmental management is more common, a high percentage of negative responses in the first four steps of the flowchart may indicate that there is not a sound plan in place to ensure EMS is undertaken, or that the plan has not been adequately communicated to the researchers themselves. Therefore, the hazard associated with poor performance at steps 1 to 4 of the EMPA has been termed ‘lack of engagement.’

The first step in the flowchart intentionally divided respondents based on their awareness of whether environmental risk should be considered prior to conducting a research project in their organization by asking if they had to undertake or read an environmental risk assessment prior to commencing work. Out of 171 respondents, 33% replied yes, 45% replied no and 22% said they were unsure. For example, of the 41% of researchers located in the UK, 37%, or 26 people, responded ‘no’, which is interesting as many academic organisations in the UK opt to implement ISO14001 to manage their environmental impact, therefore would be assumed to have an environmental risk management process in place. Overall, 45% of researchers asked replied that there was no requirement for an ERA suggesting a lack of engagement with environmental management within their organisations, perhaps due to lack of awareness of the importance of environmental management, not prioritising environmental management or lack of legislative incentives.

**Figure 3:**
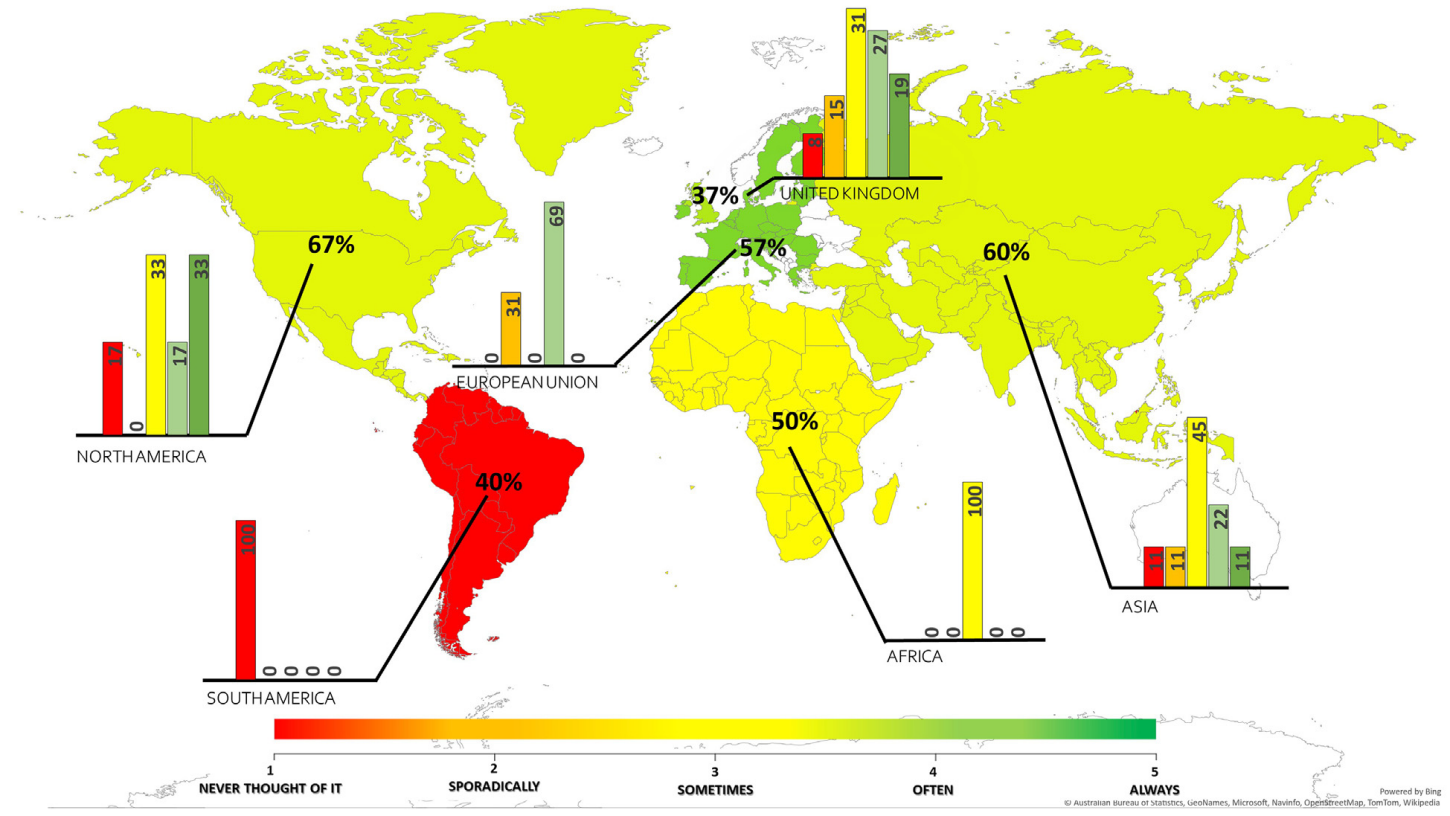
Demographic locations of participants who responded ‘no’ (% per country) when asked whether they needed to undertake/read an ERA prior to conducting research and their response to how often they personally consider the environmental impact of their research on a scale of 1–5. Where 1: never; 2: occasionally; 3: sometimes; 4: often; 5: frequently.

If respondents answered no, the survey ended as all other questions pertained to the EMS process in their organisation. However, before exiting, data was gathered on how often they personally considered the environmental impact of their own research (step 2) and their geographical location (step 3). Of the 45% of researchers who responded ‘no’ to step 1, almost half said that on a scale of 1–5 they frequently i.e., every time they started a project, or every time they worked in a laboratory, thought about the environmental impact of their research suggesting many researchers would be receptive to implementation of a formal EMS process (Figure 3). The consequence of not assessing environmental risk may lead to unexpected environmental impacts that require reactive remediation, which may be costly, incur environmental penalties and ultimately cause damage to the reputation for the entire organisation. This could result in potential collaborators perceiving the organisation as being negligent reducing income gain from possible work and international collaboration. Therefore, in the EMPA flowchart responding ‘no’ to step 1 result in an ‘engagement’ hazard endpoint.

Of the 22% of researchers who responded ‘unsure’ to step 1, 18% of these researchers replied that there was no ERA or awareness training within the organisation (step 4), also suggesting that the organisation is not engaging with an environmental management process therefore leading to an engagement hazard endpoint. A further 41% of the researchers who responded ‘unsure’ to step 1 were required to undertake environment awareness training (step 4), which should inform them whether a lab-based ERA is required. The final 41% were not aware of whether any training was provided. Both responses suggest that the organisations are not communicating the requirement effectively to their researchers, which may lead to a lack of inertia to investigate further. Therefore, if there are a large percentage of negative (‘no’ or ‘unsure’) responses to step 4 the hazard is ineffective communication, which could result in significant environmental impact if organisations are unaware of the risk research poses to the environment, or if researchers do not know how to manage an incident that has an environmental impact.

Steps 5 to 7 of the EMPA flowchart align to the ‘do’ phase of the Deming cycle with survey questions focused on the specific processes involved in ERA to determine whether researchers undertake and are fully aware of the process. The 33% of respondents who replied affirmatively to having an ERA process (step 1), were asked to indicate whether they were aware of who was responsible for carrying out the ERA at their organization (step 5) i.e., do the researchers complete it themselves or is it done on their behalf by the university. In this case, 75% of respondents who reached this step gave a defined role as an answer (32% the supervisor, 36% the researcher (either staff or student), 16% the laboratory manager, 13% technical staff and 3% multiple named roles), and these responses were combined as ‘positive’. The remaining 25% were unsure or did not know who the responsible person within the organisation was, and these were grouped as a ‘negative’ response. As expected, the 25% of respondents who were unsure, also did not know who to report to if an accident occurred (step 6) justifying the route in the flowchart from step 5 and step 6 to the ‘communication’ hazard via step 4. On the other hand, if a respondent answered ‘no’ to whether their organisation provides training (step 4), the resulting hazard is ‘lack of engagement’ as it suggests that the organization does not have a sufficient plan in place to enable researchers to carry out an ERA.

It was evident from responses that even when environmental training is in place, processes may not be effectively communicated, for example, of all the positive responses to step 4, there were 23% of respondents who were not aware that their ERA needed authorization or approval, and 16% did not know who they should report to if an incident occurred. In addition, 20% were unsure whether they had to do an ERA for their project at all. This may be because training is not mandated in all universities, or it may be because training is not renewed regularly and therefore researchers are not aware of policy updates or changes in responsibilities. It may also be due to issues with the training package itself, for example online generic training may not be as effective as live face to face training, and training varies widely between universities as there is no standard EMS process.

The 75% of researchers who responded affirmatively to step 5 (responsible person), also knew who to report to if an environmental incident occurred (step 6). In the survey, respondents were then asked whether their ERA required authorisation (step 7) to determine whether their organization has an assurance process in place e.g., review by a subject matter expert. Within the EMS process, this would ensure that the appropriate processes are followed, such as disposal methods. The responses to this question were combined with responses to whether the ERA was reviewed regularly as both these questions imply that the EMS process is monitored by the organisation moving from the ‘do’ phase of the Deming cycle to the ‘check’ phase. Regular review ensures there is no ‘activity drift’ i.e., the process is being carried out as originally intended. While, if no review process is in place, outdated procedures may continue long after university regulation and policy has changed. For example, in response to legislation resulting in non-compliant activities being carried out. However, for the 19% of respondents who reached step 7, environmental impacts are being considered for their research and that they are aware of the university processes. Therefore, negative responses at step 7 and step 8 suggest there is no feedback mechanism within the university. Without effective monitoring through authorisation and review, activity drift or outdated procedures may result in unintended environmental impacts. Whilst the impacts are likely to be much less severe than if there is no mitigation in place it is possible that minor legislative non-compliance could result in penalties from authorising bodies. Therefore, the hazard at this stage has been defined as ‘no feedback mechanism,’ indicating that environmental management is weak at the ‘check’ stage of the Deming cycle. By this stage in the EMPA flowchart 94% of all respondents to the survey had been filtered out, alluding that globally many researchers are not involved in effective environmental risk management.

The final question in the survey asked the researchers whether their university had a system that enabled the identified environmental impacts to be shared throughout the organisation to ascertain whether the university could consolidate common environmental impacts for mitigation. Having a mechanism to share common impacts and mitigations would enable an organisation to identify holistic mitigation solutions, and potentially implement universal policies that can be communicated back to researchers e.g., through training. This completes the first iteration of the Deming Cycle, which should now be repeated to ensure continual improvement. When there is no mechanism for reviewing cross-university environmental impact it is unlikely that effective strategies for improvement can be implemented. Therefore, negative responses to step 9 were filtered to the ‘no continual improvement’ hazard. Where it is evident that processes are in place to ensure continual improvement (positive responses to step 9), the organisation is directed to the start of the flowchart. This is to emphasize that the EMPA flowchart provides a snapshot in time of the efficacy of the current EMS processes. Therefore, the organisation should regularly repeat the self-assessment so that the plan-do-check-act environmental management process can be monitored for continual improvement. Interestingly, only 11% of the researchers surveyed were not filtered out by step 9, revealing that environmental management is inconsistent across the research laboratories surveyed.

EMPA can be used within an organisation as a self-assessment mechanism to evaluate the efficacy of the environmental management processes. This would be achieved by conducting an internal survey of researchers to quantify the percentage of responses to targeted questions about environmental risk management to identify where processes are inefficient. Four hazards were identified linked to the four phases of the Deming Cycle (plan-do-check-act): Lack of engagement; ineffective communication, no feedback mechanism, and no continual improvement respectively. The EMPA flowchart can then be used to map staff responses to determine the hazard by reviewing the percentage of staff filtered out at each step. It is then up to the organisation to determine the percentage of negative responses that would be classed as significant, e.g., if 30% of staff are unaware who to report an environmental incident to, it is up to the organisation to determine if this needs to be improved.

**Figure 4:**
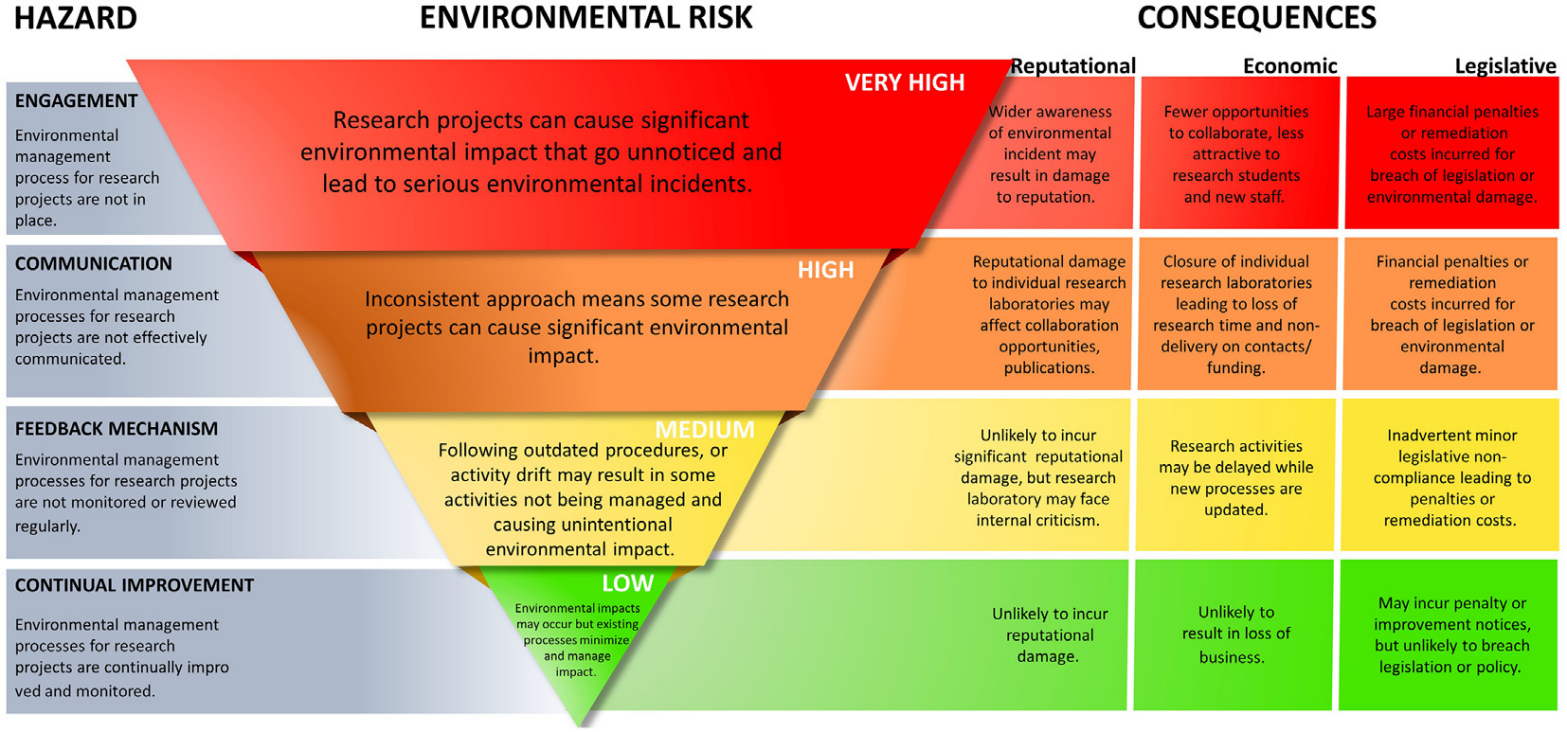
Environmental Management Performance Assessment hazards aligned to reputational, economic, and legislative consequences based on levels of environmental risk.

However, not all hazards result in equally significant environmental risks as the risk reduces dependent on the maturity of the EMS e.g., if an organisation does not require researchers to complete an ERA there is a high likelihood that an incident will occur resulting in significant consequences. However, if all researchers in an organisation are aware of the EMS process, and environmental impacts are effectively managed then the likelihood of a significant environmental impact occurring is much lower. Figure 4 summarises the identified hazards and the wider consequences that may result from ineffective environmental management in research laboratories as discussed throughout this paper. The wider consequences were selected based on common academic values such as high research impact and quality which were grouped into reputational, economic and compliance themes to reflect the potential consequences for an organisation at each of the four hazards. It follows that hazards that occur early in the Deming Cycle (lack of engagement and ineffective communication) have a very high environmental risk and therefore have significant consequences for the entire organisation, while hazards that occur later in the Deming Cycle (no feedback mechanism and lack of continual improvement) have a much lower environmental risk, with less significant and more localised consequences (Figure 4).

## 4 Conclusion

Managing environmental risk is essential to ensure organisations minimise their impact on the environment, comply with environmental legislation and maintain their reputation in an increasingly environmentally aware society. To do this, organisations usually use a management system based on the plan-do-check-act methodology. However, currently there is no method for an organisation to assess whether employees are aware of implemented environmental management systems, nor the level of conformity with environmental management processes. Therefore, this paper introduces EMPA as a novel approach to identify the level of risk to an organisation from inefficient application of environmental management process. As summarised in Figure 4, EMPA identifies four potential hazards associated with the four exit points: 1) ‘engagement’, where employees are unaware of the existence of an environmental management system; 2) ‘communication’, where key processes are not effectively communicated to employees; 3) ‘feedback mechanism’, where the efficiency of processes is not monitored and 4) ‘continual review’ where feedback is not used to implement improvements to the EMS. EMPA is carried out by surveying employees and aligning responses with the flowchart to identify the hazards, the higher the percentage of responses exiting at one of the four hazards, the higher the likelihood of the hazard being realised. An organisation with a well performing EMS would expect 100% of responses to complete the flowchart demonstrating low risk of significant environmental impacts.

To demonstrate the EMPA process a question set was created and shared on-line in the form of a voluntary survey requesting responses from academic laboratory researchers around the world. Based on the global survey, 39% of respondents exited the survey at the ‘planning stage’ identified as a ‘engagement’ hazard, this was expected as globally many nations do not have stringent environmental regulation nor the requirement for environmental management processes. No respondents were able to complete the survey, suggesting that in countries where environmental management is considered important, not all academic institutions are effectively implementing all elements of the Deming Cycle. A low percentage of respondents (9% and 6%) continued to the ‘Checking’ and ‘Acting’ phases suggesting employees are unaware of the feedback mechanisms required to improve the mitigation and monitoring processes, or that continual improvement mechanisms are not functioning. Though this presents a low-level risk to the academic institution, it highlights that even organisations with effective EMS can benefit from the EMPA process to target specific areas for improvement.

While the survey used to demonstrate the EMPA process in this paper was designed to be applicable to a wide range of academic research laboratories, however when applying EMPA within a single organisation the question set should be modified to include organisation-specific processes. By doing this, EMPA enables rapid baselining of employee awareness and conformity to environmental management processes and identification of specific areas to target for improvement through an organisation's employees.

## Declarations

### Author contribution statement

M. Ladyman, E. Gutierrez-Carazo, F. Persico & T. Temple: Conceived and designed the experiments; Performed the experiments; Analyzed and interpreted the data; Wrote the paper.

F. Coulon: Analyzed and interpreted the data; Wrote the paper.

### Funding statement

This work was supported by 10.13039/501100000859Cranfield University.

### Data availability statement

Data associated with this study has been deposited at CORD under the accession number 10.17862/cranfield.rd.14217197.

### Declaration of interests statement

The authors declare no conflict of interest.

### Additional information

No additional information is available for this paper.
