# Supplementary material for: Assessing the performance of environmental management in academic research laboratories
Source: Heliyon. 2022 Mar 21;8(3):e09135. doi: 10.1016/j.heliyon.2022.e09135 (PMC9280376; doi:10.1016/j.heliyon.2022.e09135)
Supplement: Supplementary [file mmc1.docx]

**Assessing the performance of environmental management in academic research laboratories**

M. Ladyman ^a^, E. Gutierrez-Carazo ^a^, F. Persico ^a^, T. Temple ^a^, F. Coulon ^b^

^a^ Cranfield University, Centre for Defence Chemistry, Defence Academy of the United Kingdom, Shrivenham SN6 8LA, UK

^b^ Cranfield University, School of Water, Energy and Environment, Cranfield MK43 0AL, UK

Supplementary: Full Qualtrics Survey

Assessment of environmental risks for proactive laboratory management.

Start of Block: Part 1.

Q1 Researchers at Cranfield University request your participation in a study into environmental risk assessment procedures in university teaching and research laboratories. By taking part in this survey you: 
1. confirm you currently or have previously worked or studied in research labs at an academic institution; 
2. confirm that you have been informed about this research project and agree to take part;
3. understand that the data provided will be used by Cranfield University for the purpose of research. The data will be stored on the University’s network that can only be accessed by authorised users, in line with UK Data Protection Act 2018;  
4. understand that the information I provide is anonymized and will not be connected with me in any way and therefore, once submitted, cannot be removed or deleted; 
5. understand analytical software will be used to aggregate the results of the research and  data will be published in support of the research findings.

Participation in this study is completely voluntary. If you decide not to participate there will not be any negative consequences. Please be aware that if you decide to participate, you may stop participating at any time and you may decide not to answer any specific question.”

I grant permission for the data generated from this interview to be used in the researcher's publications on this topic.

- Yes (1)
- No (2)

Skip To: End of Survey If Q1 = No

Skip To: Q23 If Q1 = Yes

Q23 In this survey Environmental Risk is defined as the “actual or potential threat of adverse effects on living organisms and the environment by effluents, emissions, wastes, resource depletion, etc., arising out of an organisation's activities.”

Q2 Where is your academic institution?

- United Kingdom (1)
- European Union (2)
- North America (3)
- South America (4)
- Africa (5)
- Australia and New Zeland (6)
- Asia (7)
- Prefer not to say (8)

Q3 What is/was your role?

- Undergraduate student (1)
- Post-graduate student (2)
- Mature student (3)
- Technical staff (4)
- Post-doctorate researcher (5)
- Research fellow (6)
- Principal investigator (7)
- Other (please state) (8) ________________________________________________

Q4 What is your research area?

________________________________________________________________

Q5 On a scale of 1-5 how often do you consider the impact of your research on the environment?

- 1 - Never thought of it (1)
- 2 - Sporadically (2)
- 3 - Sometimes (e.g. at the outset and when there are changes) (3)
- 4 - Often (4)
- 5 - Almost always when doing research (5)

Q6 Does your organization provide environmental management training?

- Yes (1)
- No (2)
- Unsure (3)

Q7 Does your institute require you to fill out or read a formal Environmental Risk Assessment before starting an experiment/procedure?

- Yes- been informed about an environmental risk assessment for my research (1)
- Yes- read and agreed to an environmental risk assessment for my research (2)
- Yes- conducted environmental risk assessment for my research (3)
- No (4)
- Unsure (5)

Skip To: Q8 If Q7 = No

Skip To: Q9 If Q7 = Yes- been informed about an environmental risk assessment for my research

Skip To: Q9 If Q7 = Yes- read and agreed to an environmental risk assessment for my research

Skip To: Q9 If Q7 = Yes- conducted environmental risk assessment for my research

Skip To: Q9 If Q7 = Unsure

Q8 Do you know why not?

________________________________________________________________

Skip To: End of Survey If Condition: Do you know why not?  Is Not Empty. Skip To: End of Survey.

Skip To: End of Survey If Condition: Do you know why not?  Is Empty. Skip To: End of Survey.

Q9 What type of environmental risk assessment process is undertaken at your institute?

- Qualitative matrix based (e.g. frequency x severity) (1)
- Quantitative (e.g. computational derivation of severity/ frequency) (2)
- Both Qualitative and Quantitative (3)
- Free form (e.g. hazard assessment) (4)
- Other (please state) (5) ________________________________________________
- If you have time, please provide more details about your risk assessment type (6) ________________________________________________

Q10 In your institute, who is responsible for completing or undertaking the environmental risk assessment?

- The researcher (student or staff) (1)
- The supervisor (2)
- The laboratory manager (3)
- Technical staff (4)
- Unsure (5)
- Other (please state) (6) ________________________________________________

Q11 Does your environmental risk assessment require authorisation or approval?

- Yes (1)
- No (2)
- Unsure (3)

Q12 How are environmental risk assessments stored at your institution?

- Electronically within research group (1)
- Hard copy (2)
- Hard copy and electronically (3)
- Cross-institution database or repository (4)
- Unsure (5)
- Other (please state) (6) ________________________________________________

Q13 How often are environmental risk assessments reviewed at your institution?

- Less than 6 months (1)
- Annually (2)
- Every two years or more (3)
- Never (4)
- Unsure (5)
- Other (please state) (6) ________________________________________________

Q14  If an environmental incident or accident occurred in your research laboratory, do you know who to report to?

- Yes (1)
- No (2)
- Unsure (3)

Q15 Does your institute provide training for how to write or conduct an Environmental Risk Assessment?

- Yes (1)
- No (2)
- Unsure (3)

Q16 In your opinion, what is the most significant environmental impact of your research?

- Energy use (1)
- Water use (2)
- Material resource use (3)
- Chemical waste generation/ disposal (4)
- Biological waste generation/ disposal (5)
- Air/land/water pollution (6)
- Other (please state) (7) ________________________________________________

Q17 Do you think the environmental risk assessment processes in place at your institution satisfactorily prevents or minimizes the impact on the environment from your research?

- Yes (1)
- No (2)
- Prefer not to answer (3)

Skip To: Q18 If Q17 = No

Skip To: End of Survey If Q17 = Yes

Skip To: End of Survey If Q17 = Prefer not to answer

Q18 If no, please let us know how you think the environment risk assessment process could be improved.

________________________________________________________________

End of Block: Part 1.
